# Discontinuation of anti-hypertensive drugs increases 11-year cardiovascular mortality risk in community-dwelling elderly (the Bambuí Cohort Study of Ageing)

**DOI:** 10.1186/1471-2458-14-725

**Published:** 2014-07-16

**Authors:** Maria Lea Correa Leite, Joselia OA Firmo, Antonio Ignacio Loyola Filho, Maria Fernanda Lima-Costa

**Affiliations:** 1 Institute of Biomedical Technologies, National Research Council, Via Fratelli Cervi 93, 20090 Segrate, MI, Italy; 2 Rene Rachou Research Center, Oswaldo Cruz Foundation, Av. Augusto de Lima 1715, 30190-002 Belo Horizonte, MG, Brazil; 3 Federal University of Minas Gerais, Belo Horizonte, MG, Brazil

**Keywords:** Hypertension, Antihypertensive drugs, Mortality risk, Elderly

## Abstract

**Background:**

Hypertension remains a major public health problem whose management is hampered by poor persistence with pharmacological therapy. The aim of this study was to evaluate the association between discontinuing antihypertensive drugs (AHDs) and the risk of cardiovascular mortality in the elderly.

**Methods:**

A population-based prospective cohort study of all of the ≥60-year-old residents in Bambuí city (Brazil) enrolled 1606 subjects (92.2%), of whom 1494 (93.0%) were included in this study. The use of AHDs was ascertained annually in a real-clinical context, and time-varying AHD exposure was categorised as non-use, current use or stopped. The predicted cardiovascular mortality rates were estimated using interval Poisson models for ungrouped person-time data, taking into account current levels of systolic blood pressure (BP).

**Results:**

The overall adjusted cardiovascular mortality risk ratio of AHD stoppers *vs* current users was 3.12 (95% CI: 2.35-4.15). There was a significant interaction with BP levels: the association between discontinuing AHDs and the risk of cardiovascular mortality was stronger at higher systolic BP levels. The estimates of the risk of cardiovascular mortality over the follow-up period were similar in AHD users and non-users, for whom AHDs were never prescribed.

**Conclusion:**

Discontinuing AHDs increases the risk of cardiovascular mortality in the elderly. Misconceptions about symptoms or drug-related adverse effects could underlie a subject’s decision to discontinue AHDs. Greater attention should be paid to the choice of AHDs and informative action.

## Background

There is growing evidence that antihypertensive treatment is beneficial in the elderly [1], but hypertension remains a major public health problem whose prevalence is increasing throughout the world with the ageing of the population [2]. The World Health Organization (WHO) estimated that at least 50% of cardiovascular diseases and 75% of strokes result from high blood pressure (BP) [3]. Because of its high prevalence and severe consequences, efforts have been made worldwide to increase detection and control of existing arterial hypertension [4-6]. However, population-based cross-sectional studies have shown that the prevalence of appropriate control of arterial pressure (that is, systolic and diastolic BP below 140 and 90 mmHg, respectively) among individuals treated with antihypertensive drugs ranges from as little as about 40% in the USA [7], Mozambique [8] and Brazil [9] to 66% in Canada [10].

The adequate control of hypertension is seriously hampered by poor persistence with pharmacological therapy [11], particularly among the elderly [12]. There are a number of published studies concerning the predictors of persistence [13,14], and some data concerning the health and economic consequences of non-adherence [15,16] but, to the best of our knowledge, no attempt has yet been made to quantify the impact of discontinuing antihypertensive drugs (AHDs) on the risk of cardiovascular (CV) mortality in the elderly.

In Brazil, although free and widespread access to essential medicines standardized by the Unified National Health System (SUS) is constitutionally guaranteed, it is still a challenge to ensure complete therapeutic assistance. In 1998, the government approved the National Medicines Policy [17] in an attempt to promote access to and the rational use of medicines. In 2002, the National Program of Pharmaceutical Assistance to Arterial Hypertension and Diabetes Mellitus was instituted in order to provide access to essential medicines within the SUS [18]. However, a lack of free access to hypertension medicines has been reported to be mainly due to the unavailability in the public sector [19]. Therefore, in 2004, the federal government introduced the “Farmacia Popular do Brasil“ Program, establishing a co-payment scheme as a strategy for improving access to medicines. People have consistently resorted to this program to obtain essential medicines that they have been unable to access in the public sector [20].

The aim of this study was to evaluate the association between the discontinuation of AHD use and the long-term risk of CV mortality in a large population-based elderly cohort in Brazil. The Bambuí Cohort Study of Ageing dataset provided annual verification of AHD use and certified deaths along 11 years, and therefore offers a valuable means of examining the relationship between AHD exposure and mortality risk. Furthermore, the availability of data concerning other variables, including repeated BP measurements, makes it possible to control for different socio-demographic, clinical, biological and lifestyle conditions.

## Methods

### Study design and population

The data come from the Bambuí Cohort Study of Ageing, which involved a population-based cohort of older adults in Bambuí, a city of approximately 15,000 inhabitants in south-eastern Brazil. The main objective of the cohort study was the examination of the consequences of the double burden of non-communicable diseases and chronic Chagas’ disease, an infection caused by the protozoan *Trypanosoma cruzi *[21,22]. The study procedures have been described in detail elsewhere [21]. Briefly, the baseline cohort consisted of 1742 residents aged ≥60 years on 1 January 1997, who were identified by means of a complete census in the city. Of these, 1606 (92.2%) participated in the baseline survey, which consisted of a questionnaire designed to collect data relating to socio-demographic variables, lifestyles, medical history and drug use, laboratory tests (biochemical and hematological analyses), and physical measurements (BP and anthropometric measures). All of the interviewers and health technicians were certified after training at a specialized center at the School of Medicine of the Minas Gerais Federal University. The mean age of the participants was 69.3 years (range 60–95 years), and 60.0% were women. The follow-up was based on annual interviews and the verification of death certificates, and collected information concerning the current use of medication.

The participants signed an informed consent form and authorised the verification of death certificates. The Bambuí Cohort Study of Ageing was approved by the Ethics Committee of the Fundação Oswaldo Cruz, Brazil.

### Source of mortality data

The analysis was based on the deaths occurring between the time of study enrolment and 31 December 2007. The deaths were reported by the next of kin during the annual follow-up interview, and confirmed by the Brazilian Mortality Information System (Sistema de Informações sobre Mortalidade) with the permission of the Brazilian Ministry of Health. Death certificates were obtained for 98.9% of the subjects. Deaths due to CV causes were defined as those caused by hypertensive diseases, ischemic heart diseases, pulmonary heart disease, cerebrovascular diseases, diseases of arteries, arterioles and capillaries, and cardiovascular involvement in chronic Chagas’ disease.

### AHD exposure

The current use of AHDs was ascertained annually by reviewing medication containers and/or physician prescriptions during home visits to all of the participants. The medications were coded on the basis of the Anatomical Therapeutic Chemical/daily drug dose (ATC/DDD) classification system [23], and were considered AHDs if they fell into ATC groups C02 (anti-hypertensives), C03 (diuretics), C07 (beta-blockers), C08 (calcium channel blockers), or C09 (agents acting on the renin-angiotensin system). Time-varying AHD exposure was estimated by dividing the follow-up into mutually exclusive periods of non-use (no current or previous prescription), current use, and stopped use (non-use after a previous prescription) for each subject.

### Blood pressure measurements

Systolic and diastolic BP were measured at baseline (1997), and after three (2000), five (2002) and eleven (2008) years of follow-up, using standard desk mercurial sphygmomanometers (Welch Allyn Tycos 5097–30, Tycon, Arden, USA) and stethoscopes (Littman Cardiology II, 3 M Medical Devices, St. Paul, USA). Depending on the subject’s right arm circumference, a standard (12×23 cm) or large adult cuff size (15×32 cm) was used. At each time point, three measurements separated by 2-minute intervals were made after an initial 5-minute rest, and 30 minutes or more after the last caffeine intake or last cigarette. The measurements were made in the early morning in a quiet, isolated, temperature-controlled room at the project field clinic by appropriately trained technicians, with the subjects seated with the arm supported at heart level. The recorded BP was the arithmetic mean of the second and third measurements. Current BP was calculated by means of the linear interpolation of two consecutive measurements.

### Other variables

In addition to gender, age, educational level, conjugal status and monthly family income (measured in number of Brazilian minimum wages), we included baseline measures of waist circumference, smoking (with current smokers being defined as those who had smoked at least 100 cigarettes during their lifetime and were still smokers), alcohol consumption (with a high intake being defined as an average of more than two drinks per day for men and more than one drink per day for women during the previous 12 months), history of coronary heart disease, self-rated health (as assessed by the answer to the question “*How would you rate your own health?*”), and plasma levels of triglycerides, LDL-cholesterol, HDL-cholesterol, fasting glucose, uric acid and C-reactive protein. The blood samples were drawn after a recommended 12-hour fast, and the plasma measurements were made using standard enzymatic methods (Merck KgaA, Darmstadt, Germany).

### Statistical analysis

The data were fitted using interval Poisson models for ungrouped person-time data [24]. To this end, an analytical data set was constructed in which each subject contributed multiple observations, each observation representing one unit (a semester) of person-time. This approach avoided the need to categorise the variables originally measured on a continuous scale (such as the BP measurements) as well as dynamically accommodated time-dependent variables. The Poisson regression method is similar to Cox’s proportional hazard regression analysis and produces equivalent risk estimates [25], but is more suitable when working with mortality rates rather than survival curves. Poisson models directly estimate adjusted rate ratios (RRs) and make it possible to obtain predicted mortality rates (MRs), thus allowing the calculation of cumulative mortality rates (CMRs) for the 11-year period. The two measures have different meanings: the RRs and MRs indicate the force of mortality, whereas the CMRs directly estimate the average risk (probability) of death for a member of the cohort over the period. Robust estimators of standard errors [26] were used, and the models included terms for time-dependent BP and age (both updated half-yearly), AHD exposure (updated yearly), and all of the baseline variables mentioned above. As a preliminary separate analysis of men and women did not reveal any substantial between-gender differences, we subsequently pooled the data while keeping gender as a control variable.

The analyses were made using the Generalized Linear Models procedure of Stata software, version 11.2 [27].

## Results

Of the 1606 subjects enrolled, 1494 (93.0%) had their BP measured at baseline and were included in the analysis. Table 1 shows the baseline characteristics of the study subjects.

**Table 1:** Baseline characteristics of the 1494 study subjects

Women, n(%)	908 (60.8)
Mean age, years (range)	69.1 (60–95)
Educational level < 4 years, n(%)	962 (64.4)
Conjugal status, n(%)	
Married/live together	735 (49.2)
Never married	159 (10.6)
Divorced/separated	77 (5.2)
Widowed	523 (35.0)
Monthly family income*, n(%)	
< 2	453 (30.3)
2 – 3.9	555 (37.2)
≥ 4	486 (32.5)
Current smokers, n(%)	269 (18.0)
Alcohol consumption, n(%)	
Teetotallers	680 (45.5)
Ex-consumers	492 (32.9)
Low/moderate consumers	179 (12.0)
High consumers	143 (9.6)
History of coronary heart disease, n(%)	163 (10.9)
Self-rated health, n(%)	
Very good	41 (2.7)
Good	319 (21.4)
Reasonable	734 (49.1)
Bad	400 (26.8)
Mean waist circumference, cm (SD)	91.2 (11.1)
Mean LDL-cholesterol level, mg/dL (SD)	154.8 (44.9)
Mean glucose level, mg/dL (SD)	108.6 (43.3)
Median triglyceride level, mg/dL (IQ range)	130.0 (91.0-183.0)
Mean HDL-cholesterol level, mg/dL (SD)	49.2 (15.1)
Mean uric acid level, mg/dL (SD)	5.3 (1.7)
Median C-reactive protein level, mg/L (IQ range)	3.3 (1.5-6.7)
Anti-hypertensive drug use, n(%)	736 (49.3)

SD: standard deviation; IQ: interquartile.

*Measured in number of Brazilian minimum wages.

The mean duration of follow-up was 8.7 years, and 574 deaths (of which 240 from CV causes) occurred. There were few losses to follow-up during the eleven years from 1997 to 2007 (5.2%), leading to 13055.1 person-years (py) of observation. The overall estimated CV MR was 18.2‰ py (95% confidence interval [95% CI]: 15.9-20.5‰ py).

**Figure 1:**
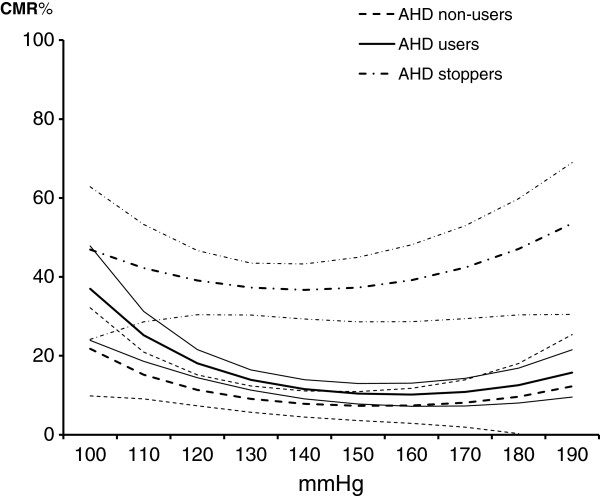
Cardiovascular cumulative mortality rates (CMR) and 95% confidence intervals by systolic blood pressure levels and antihypertensive drug (AHD) exposure.

Table 2 shows the observed CV deaths and person-years at risk, and gives the estimated MRs and RRs (with the AHD users as the reference group) stratified by systolic BP levels. The overall adjusted RR of CV mortality estimated for the AHD stoppers was 3.12 (95% CI: 2.35-4.15). The MRs were generally (but not significantly) lower in the AHD non-users group compared with the current users.In order to clarify further the relationship between mortality risk and systolic BP levels in the three AHD exposure groups and evaluate interactions terms, we ran the model including systolic BP as a continuous variable. As simple graphic inspection of the raw data suggested a non-linear relationship between MRs and systolic levels, linear and quadratic terms for continuous BP levels were included in the model. The quadratic terms were statistically significant (p-values <0.02) in all of the three groups. Figure 1 shows the CMRs obtained from the expected MRs and their 95% confidence intervals plotted in relation to systolic BP levels. The CMRs indicate the mean individual risk (or probability) of CV death over the 11-year period. U-shaped curves (negative linear terms and positive quadratic terms) related CV mortality to systolic BP levels in all three exposure groups. The subjects in the AHD users group had a 37.0% probability of CV death if their systolic BP was 100 mmHg, the CMR fell to 10.2% at 160 mmHg and then rose slowly to 15.7% at 190 mmHg, and a similar curve describes the variation of the CV CMRs in the AHD non-users group. However, the AHD stoppers had a 46.9% probability of CV death if their systolic BP was 100 mmHg, a 36.7% probability at 140 mmHg, and a 53.6% probability at 190 mmHg: the p-values for the interactions were 0.027 (linear term) and 0.045 (quadratic term).

**Table 2:** **Person**-years (PY), cardiovascular deaths (CVd), estimated* cardiovascular mortality rates (CVR, ‰ person-years) and rate ratios (CVRR, 95% confidence interval) by systolic blood pressure levels and antihypertensive drug exposure

**Systolic blood pressure (mmHg)**	**Anti-hypertensive drug exposure**											
	**Non-users**				**Current users**				**Stoppers**			
	**PY**	**CVd**	**CVR**	**CVRR**	**PY**	**CVd**	**CVR**	**CVRR**	**PY**	**CVd**	**CVR**	**CVRR**
< 120	1019.6	17	17.3	0.66 (0.36,1.22)	1070.1	32	26.2	1.00	257.1	21	60.6	2.31 (1.35,3.97)
120 - 139	1768.4	13	8.3	0.60 (0.31,1.16)	2854.6	39	13.8	1.00	615.6	30	37.5	2.72 (1.68,4.40)
140 - 159	770.3	4	5.7	0.62 (0.21,1.81)	2501.8	22	9.1	1.00	507.2	26	42.0	4.60 (2.59,8.16)
≥ 160	276.3	3	11.9	0.75 (0.22,2.59)	1239.5	20	15.9	1.00	174.6	13	60.8	3.84 (1.91,7.72)
Total	3834.6	37	10.0	0.67 (0.44,1.01)	7666.0	113	15.0	1.00	1554.5	90	46.7	3.12 (2.35,4.15)

*Using generalised linear Poisson model with a robust estimator of standard error. The model included terms for gender, time-dependent terms for age, systolic blood pressure and AHD use, and terms for baseline educational level, conjugal status, monthly family income, smoking, alcohol consumption, history of coronary heart disease, self-rated health, waist circumference, and plasma levels of LDL-cholesterol, glucose, triglycerides, HDL-cholesterol, uric acid and C-reactive protein.

Table 3 shows the CV mortality RRs of AHD stoppers *vs* current users, estimated according to the duration of AHD discontinuation and stratified by systolic BP levels. At the lowest levels of BP, excess mortality occurred particularly early after drug discontinuation, and the higher the BP levels, the longer the duration of the discontinuation period associated with an increased mortality risk. Therefore, in those subjects exposed to AHD discontinuation for longer than one year, there were clear increasing trends of mortality risk across increasing levels of systolic BP.

**Table 3:** Estimated*cardiovascular mortality rate ratios (95% confidence interval) by systolic blood pressure levels and duration of antihypertensive drug (AHD) discontinuation

**Systolic blood pressure (mmHg)**	**Duration of AHD discontinuation**		
	**< 1 year**	**1-2 years**	**≥ 2 years**
<120	3.94 (2.12,7.35)	2.29 (0.82,6.41)	0.97 (0.35,2.66)
120 - 139	4.57 (2.53,8.25)	3.40 (1.53,7.54)	1.25 (0.55,2.80)
≥ 140	4.15 (2.19,7.88)	6.32 (3.35,11.93)	3.06 (1.64,5.69)
Total	4.27 (2.95,6.19)	4.11 (2.62,6.44)	1.75 (1.13,2.71)

*Using generalised linear Poisson model with a robust estimator of standard error. The model included terms for gender, time-dependent terms for age, systolic blood pressure and AHD use, and terms for baseline educational level, conjugal status, monthly family income, smoking, alcohol consumption, history of coronary heart disease, self-rated health, waist circumference, and plasma levels of LDL-cholesterol, glucose, triglycerides, HDL-cholesterol, uric acid and C-reactive protein. Current AHD users are the reference group.

## Discussion

Hypertension is one of the leading clinical conditions affecting the elderly; it increases their risk of cardiovascular, cerebrovascular and renal diseases, and requires long-term treatment (often for an entire lifetime). It is widely acknowledged that treating hypertension in the elderly is important as it improves their quality of life and lowers the incidence of complications [28], but ensuring the continuation of long-term drug therapy over time is a considerable public health challenge, especially among older people. Our findings indicate that, in comparison with their currently treated counterparts, elderly subjects who discontinue AHDs are at three times higher risk of death due to CV diseases.

The strength of our study mainly lies in its prospective nature and the fact that it involved a large number of community-dwelling elderly over a long period. As one of its characteristics was its real-world context, the time-dependent AHD exposure should reflect real clinical dynamics. The study is further strengthened by the fact that the availability of repeated BP measurements allowed us to adjust for the severity of hypertension and obtain optimal estimates of individual current BP levels during the follow-up period, thus overcoming the two problems of indication and dilution biases. Unlike other studies that have measured persistence with therapy using administrative health databases [29], our data were based on the direct verification of drug containers and/or prescriptions, and the uniformity of the data collection procedures assures comparability between subjects. Nevertheless, given that the data were collected annually, there may have been some degree of non-differential misclassification of the time of AHD exposure. Furthermore, we did not consider medication-taking behaviour, understood as adherence to the prescribed dosing regimens, and so it is possible that some of the subjects classified as AHD users were not very compliant. However, although such potential misclassifications can introduce a bias, it should always be in the direction of underestimating the effect [30]. It is moreover worth noting that changes in public health policy (that taken place during the study period) aiming to improve access to medicines might have influenced the use and persistence following prescription of AHDs.

Our analyses were extensively controlled for potential confounding factors, including self-rated health. This variable represents a key measure of health status and is a well-established universal predictor of mortality [31]. The perception of one’s own health is presumably an important determinant of health-related behaviours. In the Bambuí cohort population, the variable “self rated health” has been found to be multidimensional in structure, reflecting socioeconomic conditions, social support, health status and access to/use of health services [32]. We don’t know the specific reasons for treatment discontinuation; however, a previous qualitative study of the beliefs and behaviour of the hypertensive elderly people in the Bambuí cohort showed that the absence of symptoms led many of them to feel they were not ill and, accordingly, they underestimated the importance of continuing to use their medications [33]. In addition, an ethno-epidemiological inquiry, conducted with the aim of comprehending hypertension-related experiences in the elderly population of Bambuí, showed that conflicting cultural construction of “blood pressure problems” contributed to non-adherence to treatment [34]. Furthermore, a study of the reasons given by Brazilian hypertensive patients for discontinuing pharmacological treatment showed that the most frequent was the “normalisation” of BP, followed by the side effects of the medications and forgetting to use them [35].

Deaths due to CV causes are likely to be complications of hypertension. If cultural factors such as misconceptions concerning symptoms underlie the decision to discontinue AHDs, our results highlight the importance of providing information/education in order to correct them. Furthermore, unlike the therapy stoppers, the current users had only slightly increased CV CMRs even at the highest levels of systolic BP, and this is of particular clinical and public health concern as poor BP control rates have been found in surveys of hypertensive patients in various countries [36]. The importance of distinguishing between drug-resistant hypertension and faulty adherence to medication has been stressed [37]. Our approach allowed us to make a clear distinction between these different clinical contexts and evaluate their associated CV mortality risks.

At the lowest levels of systolic BP, CV mortality was relatively increased in all of our AHD exposure groups, and it is possible that these deaths were related to advanced heart failure leading to low systolic BP, regardless of whether the subjects were treated or not. It has been previously suggested that the excess CV mortality reported in elderly subjects with low BP is due to poor CV health rather than the low BP itself [38]. This hypothesis is supported by our results showing that much of the excess mortality at the lowest levels of systolic BP arise early after AHD discontinuation.

The main purpose of allowing for the non-AHD users group was to ensure that the comparison of interest (AHD stoppers *vs* current users) has been made by including only subjects for whom AHDs was prescribed, therefore making it more meaningful. However, the risk of CV mortality in the group of non-AHD users was comparable with that of current users. It is important to remember that this group included subjects for whom pharmacological therapy was not indicated, either because they were not hypertensive or because their hypertension was clinically considered less severe, and so lifestyle modifications may have been all that was necessary to reduce the risk even at high BP levels. This hypothesis is indirectly supported by the fact that there was a high prevalence of hypertension awareness at baseline (about 80%) [9], and the cohort was subsequently well monitored. Thus, it is presumable that the physicians recommended lifestyle modifications instead of prescribing drug therapy in absence of another risk factors or target organ damage. Several mechanisms have been proposed to explain the possible protective effect of lifestyle interventions against CV diseases in the elderly, including a reduction of vulnerability. Thus, lifestyle practices (particularly physical activity and caloric restriction) could minimize the risk of death from CV disease in older persons [39]. In this concern, our results seem suggest that pharmacological treatment could be not always necessary to reduce risk. On the other hand, the findings regarding the stoppers suggest that once AHDs were prescribed, they should not be discontinued, except on medical advice.

It has been institutionally recognised that lack of persistence to long-term therapies is an international problem. Official recommendations to improve persistence include supporting patients by means of tailored multidisciplinary interventions [40]. Our data highlight the importance of cultural aspects, particularly the gap between objective and subjective judgements of the seriousness of the disease, and suggest that hypertensive patients should be helped to acquire appropriate perceptions concerning CV risk and the benefits of regularly taking their prescribed medication.

## Conclusion

Older subjects who discontinue the use of AHDs have a three times higher risk of CV mortality than those continuing treatment. Our findings highlight the discontinuation of pharmacological antihypertensive therapy in the elderly as a major public health problem and suggest the benefit of informative action aimed at giving older subjects an appropriate perception of disease symptoms and the related risk of complications.

## Competing interests

The authors declare that they have no competing interests.

## Authors’ contributions

MFLC: design and coordination of the survey, conception and design of the study, interpretation of data, critical revision of the manuscript. JOAF: acquisition of subjects and data, interpretation of data, critical revision of the manuscript. AILF: acquisition of subjects and data, interpretation of data. MLCL: conception and design of the study, statistical analysis of data, interpretation of data, drafting of the manuscript. All authors read and approved the final manuscript.

## Pre-publication history

The pre-publication history for this paper can be accessed here:

http://www.biomedcentral.com/1471-2458/14/725/prepub
